# One millimeter per minute growth rates for single wall carbon nanotube forests enabled by porous metal substrates†

**DOI:** 10.1039/c7ra13093g

**Published:** 2018-02-19

**Authors:** Naoyuki Matsumoto, Azusa Oshima, Sachiko Ishizawa, Guohai Chen, Kenji Hata, Don N. Futaba

**Affiliations:** National Institute of Advanced Industrial Science and Technology (AIST) Central 5, 1-1-1 Higashi Tsukuba Ibaraki 305-8565 Japan matsumoto-naoyuki@aist.go.jp d-futaba@aist.go.jp +81 29-861-4851

## Abstract

We report an exceptionally high-efficiency synthesis of long single-wall carbon nanotube (SWCNT) forests using porous substrates (metal meshes) in place of nonporous flat substrates. This study examined the dependence of the growth efficiency on various mesh structures, including wire diameter, aperture size, and total surface area. We demonstrated that the synthesis of SWCNT forests is highly dependent on the initial porosity as well as maintaining the open pores throughout the duration of the growth. Our results show that carbon nanotubes (CNTs) can be grown on all surfaces of the mesh in high efficiency with the optimum growth efficiency observed for a mesh porosity of ∼30%. Based on these results, we demonstrated the high efficiency synthesis of SWCNT forests (height: >3.47 mm, average growth rate: 301 μm min^−1^, and yield: 12.7 mg cm^−2^ in 10 min growth time). Furthermore, we showed that the initial growth rates exceeded 1 millimeter per minute (1000 μm min^−1^). Our results further indicate that metal meshes represent a viable alternative to nonporous flat substrates for the efficient synthesis of tall and high yielding SWCNTs.

## Abstract 2

We report an exceptionally high-efficiency synthesis of long single-wall carbon nanotube (SWCNT) forests using a porous substrate (metal meshes) in place of nonporous flat substrates.

## Graphical abstract

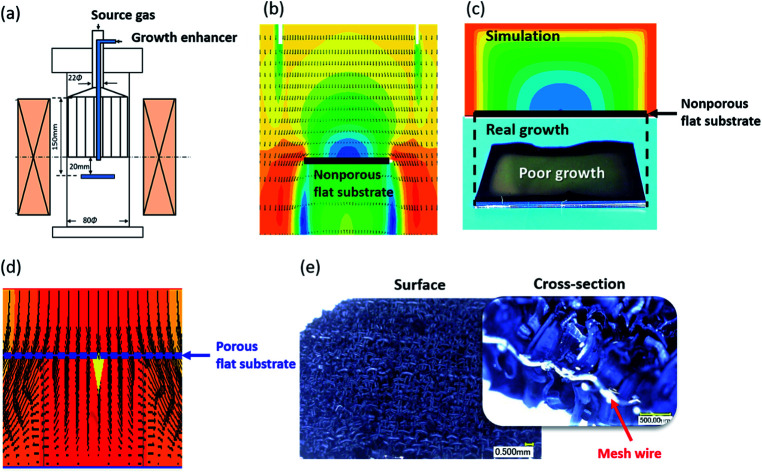

## 1. Introduction

Over the past 25 years, since the discovery of the single-walled carbon nanotube (SWCNT),^1,2^ the field has taken great strides in the development of SWCNT-based and carbon nanotube (CNT)-based applications, such as composite materials, strain sensors, and fibers.^3–5^ Utilizing their unique structural characteristics, such as diameter, density, and chirality, many of these applications benefit from their high aspect ratio, *i.e.* long length. One of the most common ways to efficiently grow long CNTs is to grow them as a vertically aligned assembly, often called a “forest”. In this way, the catalysts, which are deposited onto a substrate can grow in bulk up to the millimeter-scale,^6–8^ and have served as the basis for mass production, which is essential for the development of the CNT industry.^9^ Therefore, the development of high efficiency and large throughput methods for SWCNT synthesis is important for realizing the practical use of SWCNT applications.

Among the various methods reported to grow CNT forests, water-assisted chemical vapor deposition has shown to provide highly efficient growth of high purity and millimeter-tall CNT forests.^6^ Unique to the water-assisted approach is the exceptionally high growth rates and relatively long catalyst lifetime.^11,12^ However, an inverse relationship between the CNT forest growth rate and the catalyst lifetime has been reported using flat, nonporous substrates, which can limit the ultimate CNT forest height.^13^ The foundation of this high growth efficiency is the addition of a growth enhancer, such as water, to the synthesis ambient to maintain catalyst activity through the prevention of deactivation by carbon accumulation on the catalyst surface.^10^ The appropriate amount of growth enhancer was found to be exceptionally small, ∼1/1000th the molecular number of the carbon feedstock (ethylene).^14^ Therefore, providing a uniform delivery of this gas ratio to all catalysts across a substrate surface is of practical importance in maintaining high growth rates and uniform growth. In addition, as the scale of the flat substrate increases, the uniform delivery of this exceptionally small ratio throughout a substrate surface becomes increasingly challenging.

The fluidized bed approach for synthesis possesses the ability to overcome this challenge as the fluidization of small, substrate particles affords uniform gas delivery due to substrate mixing as well as temperature homogeneity.^15,16^ For example, Wei *et al.* developed a mass production process based on the fluidized bed method of vertical aligned CNT arrays, and is now producing CNTs on the ton scale.^15^ In addition, Noda *et al.* proposed and developed an internal heat-exchange reactor for fluidized-bed chemical vapor deposition (FBCVD) to establish a method for sub-second conversion of acetylene to sub-millimeter-long CNTs.^17,18^ Fundamental to the FB-CVD approach is the particle substrate fluidization which balances the buoyancy of the particles with that of gravity. However, as the CNT synthesis progresses, the total density of the particle/CNT decreases which results in an increase in buoyancy. For example, the growth of a ∼300 μm forest on a 500 μm particle substrate results in an 80% decrease in density. As a result, the CNT-coated particles move out of the growth region as the fluidization equilibrium position shifts upwards. Therefore, maintaining a fixed growth ambient for an extended time becomes difficult. This aspect becomes an important issue as heating of the reactive gases has shown to be essential for high efficiency synthesis. For example, Yasuda *et al.* demonstrated that the heat history (*i.e.* “dwell time”) of the carbon feedstock gas was demonstrated as a vital parameter for very rapid SWCNT forest growth with long (catalyst) lifetime.^11,19^ Therefore, a high surface area substrate system where gas can be delivered uniformly to all surfaces and held at a fixed growth condition is ideal.

Previous reports of the use of metal meshes with several surface treatments as substrates of CNT growth have shown the limited yields of multiwall carbon nanotubes (MWCNTs) by thermal chemical vapor deposition (CVD).^20–26^ These MWCNTs were grown uniformity on the surface of mesh substrates regardless of shape and size (porosity, wire diameter, aperture, and surface area), which was attributed to the source gas contact to all catalysts on the mesh substrate. We used this as a positive conceptual proof that mesh could be used for high yield SWCNT synthesis.

Here, we report an investigation of using high surface area, porous substrates to enable the high speed synthesis of SWCNT forests. Our approach was to use metal wire meshes as representative, inexpensive substrates with different wire diameter and porosity, and our results showed a clear trend between growth rates and catalyst lifetime with porosity. Macroscopically flat, but microscopically curvilinear substrates, exhibited exceptionally efficient synthesis of SWCNT forests at average growth rates exceeding 300 μm min^−1^ and a height of ∼3.4 millimeters in a 10 minutes growth time. We also clarified that the observed high growth efficiency was achieved directly as a result of the improved uniform gas impingement on all surfaces of the porous mesh structure (*i.e.* porosity and total wire surface area). Therefore, we demonstrate that a balance between these substrate structural features results in exceptionally high SWCNT growth rate and catalytic lifetime. In addition, the yield of SWCNTs produced per substrate area was found to increase by nearly 4-fold (393%) compared to flat substrates highlighting its potential. Our results further indicate that a strong dependence between yield and porosity exists to optimize CNT growth yield in contrast to the diameter or aperture size alone. Finally, we showed that through optimizing substrate porosity and the growth conditions, initial growth rates exceeding 1 millimeter per minute (1000 μm min^−1^) could be achieved. These results indicate that metal meshes represent a viable alternative to nonporous flat substrates for the efficient synthesis of tall and high yield SWCNT synthesis.

## 2. Results

### 2.1 Gas flow analysis for nonporous and porous substrates

**Fig. 1:**
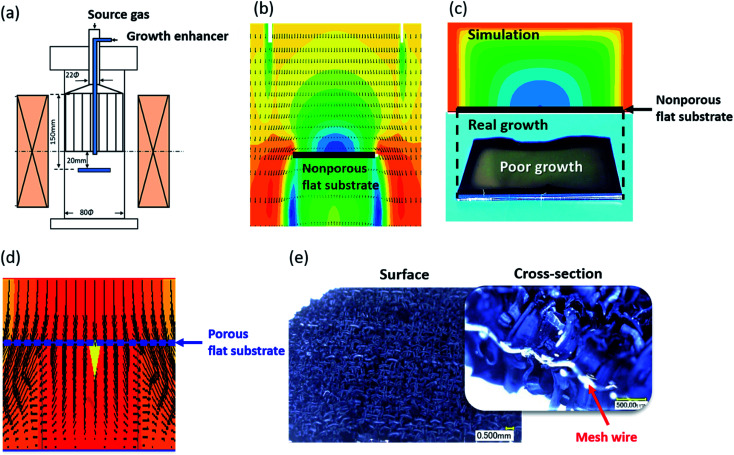
Gas flow and growth comparison between mesh and flat substrates. (a) CNT growth apparatus used in this study. Gas simulation for the CVD process for the non-porous substrate (b) side-view, (c) top-view, and (d) porous mesh substrate. (e) Grown CNTs from porous mesh substrate (inset: zoom-in of cross-section).

To begin, we performed gas flow simulations based on computational fluid dynamics (CFD) method to compare the gas flow mode for gases impinging normal to a flat, nonporous substrate and a flat, porous substrate. Gas flow simulations were carried out using PHOENICS software package (Version 2009, PHOENICS Advanced Package). Mimicking our experimental set-up of a vertical gas delivery from the gas flow rate and process temperature (Fig. 1a),^11^ our gas simulations revealed several interesting results. First, for the flat, nonporous substrate, a clear gas flow discontinuity was observed directly above and below the substrate. Second, the pressure dropped at the geometric center of the top surface resulting in a gas flux gradient across the substrate surface (Fig. 1b and c). Higher gas speeds and increased gas flux were observed at the edges of the substrate (Fig. 1b and c). These conditions can manifest in the synthesis as inhomogeneity in the forest structure, growth yield and quality as exemplified in Fig. 1c. In contrast, the gas simulation of the porous substrate showed improved gas flow continuity between the impinged surface and exiting surface. We do note that slight deviation in the exiting gas (the yellow arrow in Fig. 1d indicates the gas flow direction). In general, uniform gas flow across the entire surface was greatly improved. Specifically, like fluidized bed reactors, the gas flow was uniform across the substrate surface. However, unlike fixed bed reactors, the substrate is set at a fixed position in the reactor, and the gas flow rate can be adjusted without changes in particle fluidization. This independent control of the growth time and gas flow is advantageous for determining a process for optimizing the process time and synthesizing long SWCNTs.

For our examination, we used stainless steel mesh as representative porous substrates as they were relatively low-cost and possessed a regular pore structure. We performed a simple growth test on a porous mesh with aperture size (0.21 mm), wire diameter (0.21 mm) and porosities (25.4%) using the identical growth conditions used for flat substrates. CNTs were synthesized on both sides of metal mesh from sputtered Al_2_O_3_ (40 nm) and Fe (1.8 nm) which have been known to support the synthesis of SWCNT forests in water-assisted CVD method for flat substrates.^6^ In short, the growth was carried out using the water-assisted CVD method with a total flow of 1500 sccm, an acetylene (C_2_H_2_) carbon feedstock at 0.6% concentration, H_2_O at about 800 ppm in a helium (He) carrier gas at 815 °C in 10 min growth time at atmospheric pressure in vertical CVD furnace (Fig. 1a).^11^ The grown CNTs self-assembled into vertically oriented forests on both sides of the substrate (Fig. 1e). In accordance with the gas flow simulations, the forests were uniformly distributed across the entire 20 × 20 mm substrate with a height of ∼0.6 mm in a 10 minutes growth time. These results clearly show an increase in SWCNT growth efficiency through the use of porous substrates due to the improved uniformity of gas supply.

### 2.2 Dependence of SWCNT growth yield on substrate mesh structure

Next, in order to determine the potential of this approach, we examined the dependence of the mesh structure on the synthesis of SWCNTs. For this purpose, we utilized metal mesh substrates spanning a variety of aperture size (0.14–1.07 mm), wire diameter (0.18–0.57 mm) and porosities (19.1–71.0%). The specifications of each mesh are shown in Table 1. For simplicity and direct comparative purposes, the growth conditions, in this section, were held fixed as described in the previous section. Following the synthesis, the SWCNT forest growth efficiency were characterized by the SWCNT yield (SWCNT mass/substrate area) and forest height.

**Table 1:** Specifications of the mesh substrate in this work

Porosity (%)	Aperture (mm)	Wire diameter (mm)	Mesh (number per inch)	Relative surface area compared with flat, nonporous substrate
19.1	0.14	0.18	80	3.5
25.4	0.21	0.21	60	3.1
29.9	0.35	0.29	40	2.8
30.4	0.70	0.57	20	2.8
52.3	0.61	0.23	30	1.7
52.5	0.92	0.35	20	1.7
71.0	1.07	0.20	20	1.0

**Fig. 2:**
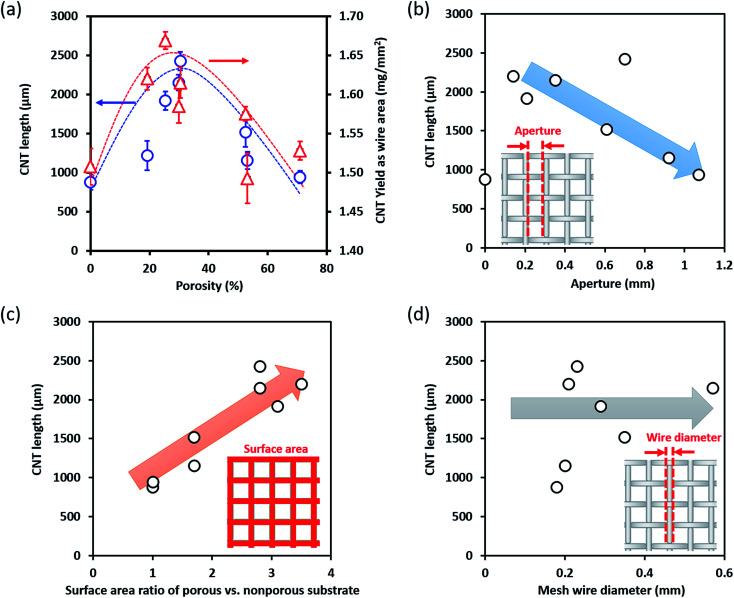
Relationship between mesh structures and SWCNT growth efficiency. (a) Porosity, (b) pore aperture, (c) surface area ratio (mesh/flat), (d) mesh wire diameter.

The calculated yield and SWCNT height were plotted as a function of the substrate porosity. As seen in Fig. 2a, as the porosity increased, the yield and SWCNT height increased, peaked at about 25.4% or 29.9%, then a steadily decreased. The result of a flat, nonporous substrate using the same catalyst and growth condition is included as a reference, which highlight the increasing trend at low porosities. As seen in Fig. 2a, the yield of CNTs grown per surface area of the substrate for the mesh is equal or higher than that of the flat substrate. We chose porosity as the index for describing the mesh structure because the wire diameter and aperture alone were insufficient. In particular, substrates can possess similar porosity (or occupancy) while possessing vastly different diameter and apertures (Table 1). To clarify this point, we investigated the dependence of wire diameter, apertures, and surface area on the grown SWCNT height (Fig. 2b–d). We found that the dependence of the height *versus* diameter did not reveal any clear trend (Fig. 2d). The height *versus* aperture and surface area did show opposing trends; the SWCNT height decreased with increase in apertures and increased with increased in surface area (Fig. 2b and c). These results indicate that while the apertures and surface area of metal mesh do show some kind of effect on the SWCNT height, they do not provide a complete description of the substrate structure.

### 2.3 Dependence of SWCNT growth kinetics on substrate mesh structure

To more quantitatively examine the dependence of the substrate mesh structure on the SWCNT growth synthesis, we investigated the growth kinetics for each variety of mesh substrate. For this purpose, we extracted the average growth rate and catalyst lifetime from each of the respective growth evolution curves as measured by *in situ* height monitoring system within the 10 minutes growth time.^27^ The lifetime was determined from fitting the measured “growth curve” to the previously reported model for SWCNT forest growth kinetics.^27^ The average growth rate was simply defined as the quotient of the height at termination and the previously determined termination time.^12,27^

**Fig. 3:**
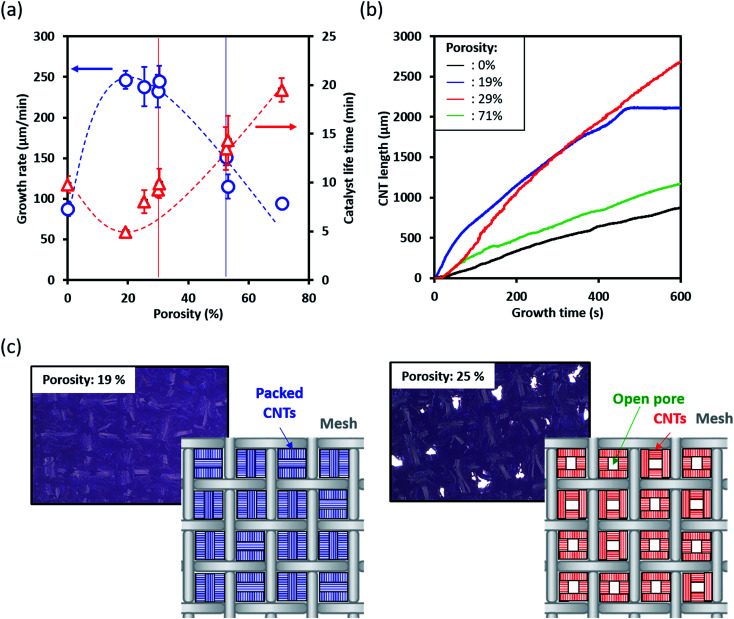
Relationship between open porosity of mesh substrate and growth mechanism. Vertical dashed lines indicate pairs of samples with similar porosities. (a) Growth rate and catalytic life time, (b) growth curves on Si substrate and mesh substrates with 19, 29, 71% porosities. (c) Digital microscope images on the top of mesh structure at 19% and 25%.

The growth rate and catalytic lifetime were then plotted as a function of porosity, and we can make several interesting observations (Fig. 3a). First, both the growth rate and lifetime were highly sensitive to changes in the porosity. Second, the growth rate and lifetime basically followed opposing trends. Specifically, as the porosity increased, the growth rate increased, peaked, and then decreased while the catalyst lifetime slightly dipped then monotonically increased. We note again that the growth conditions were fixed in this examination. Therefore, these observed changes to the growth kinetics (growth rate and lifetime) solely derive from changes to the gas flow which result from the structure of the substrate. These results highlight that the peak growth yield/height for a 10 minutes growth time stems from the combination of the highest average growth rates which can be sustained across the growth duration. Therefore, we demonstrated that porosity represents a key structural parameter related to the optimization of the SWCNT growth as it includes information on both aperture size and surface area. To highlight this point, the plot shown in Fig. 3a includes two sets of data (marked with red and blue lines, respectively) originating from substrates of similar porosities (meshes #1 and 2; meshes #3 and 4, Table 1), but different wire diameters and apertures. As observed, for each set (red and blue lines), the porosities, growth rates, and lifetimes were all similar.

While the lifetime demonstrates a clear monotonic increase above ∼30% porosity, the slight decrease in catalyst lifetime from a nonporous substrate to ∼19.1% suggests an additional process inhibiting CNT growth (Fig. 3a). As observed in the growth kinetics curves, the growth progression for the nonporous, porosity 29% and 71% are all smoothly varying and only differ by the growth rate and height (Fig. 3b). In contrast, the growth kinetics curve for the 19% porosity shown an abrupt termination. To investigate this issue, we observed the CNT growth on the low porosity (19.1% and 25.4%) meshes by digital microscopy (Keyence, VHX-5000). Please note that while both mesh substrates (19.1% and 25.4%) showed exceptionally fast growth rates, the 25.4% porosity resulted in taller forests. Observation of the meshes after growth showed that both meshes exhibited the CNT forests growing vertically away from the surface of the wire surface. Importantly it also revealed that the aperture for the 19.1% porosity mesh was completely closed by the growth of CNTs near or within the aperture spaces (Fig. 3c upper), while the aperture for the 25.4% porosity mesh remained open (Fig. 3c lower). We interpret that as the aperture closed, the gas flow changed from that observed similar to Fig. 1d for a mesh to a complex form of Fig. 1b and c but with severe localized pressure decreases and gas flow turbulence due to the “maze” of mesh-patterned vertically aligned SWCNT forest “hedges”. In short, the low porosity mesh became a highly rough, nonporous substrate. As a result, gas flow would be expected to be much poorer than the simulation results and result in stagnated carbon gases and premature catalyst deactivation due to carbon coating of the catalyst as observed in Fig. 3b.^10,11,19^ It should be noted that the resolution of the CNT forest height monitoring system has been reported previously to be ∼10 μm. From these observations, we conclude that an additional critical parameter in achieving optimum SWCNT growth yield per height was maintaining the porous structure to sustain the uniform gas flow. It should be noted that the purpose of this work is to investigate the merits of porous substrates for high efficiency SWCNT synthesis for a finite growth time. Given sufficient time and catalyst lifetime, most aperture will eventually become blocked.

In the higher porosity regions, we do observe increase lifetime but decrease in SWCNT growth rates. We attribute this simply to the reduced impingement efficiency of the carbon feedstock to the catalyst nanoparticles on mesh surface due to the larger apertures. As a result, the deactivation of catalysts due to carbon coating is reduced, hence increased lifetime and reduced growth rates.^10,11,19^

### 2.4 High SWCNT growth speed on mesh substrates

**Fig. 4:**
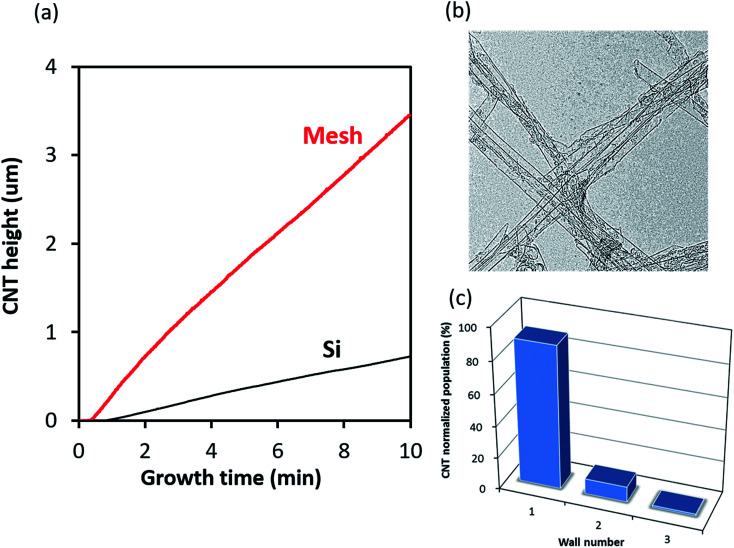
Growth efficiency of SWCNTs on mesh and Si flat substrate. (a) Growth curves of SWCNT on mesh and Si substrate, (b) TEM image of SWCNTs grown on mesh structure, (c) histogram of SWCNTs on mesh substrate from TEM image.

Finally, to demonstrate the potential of this approach, we selected the mesh which demonstrated the highest yield and then optimized the growth conditions. The flow of gases through a porous substrate represents a complex system and in addition to depending on the substrate structure (porosity), it also depends on the impinging gas flow rate. By modulating the total gas flow, temperature, carbon concentration, and water balance, the height of CNT forest was increased to over 3.4 mm in a 10 minutes growth time. The specific growth conditions were: total gas flow of 2000 sccm, acetylene concentration 1.2%, ∼1600 ppm H_2_O, and an increase in process temperature to 830 °C. As summarized in Table 2, the CNT forest growth from mesh was significantly more efficient than that for a flat nonporous substrate. In a 10 minutes growth time, the height on the mesh achieved 3.47 mm as compared with 0.88 mm for the flat substrate. As a result of the higher total surface area of the wire mesh, the total CNT yield (CNT mass per substrate size in its entirety) was 12.7 mg cm^−2^ for one side (25.8 mg cm^−2^ total, including both sides) the wire mesh and 3.2 mg cm^−2^ for the flat substrate. This represents a 3.9-fold increase, which highlights the merit of the mesh substrates for high efficiency CNT synthesis (Table 2, Fig. 4). As seen in the growth kinetics curve, the progression of the forest height is both fast and continuous without any observable termination commonly observed with high growth rate synthesis. The average growth rate was estimated to be 301 μm per minute which represents one of the highest growth rates reported. In addition, for comparison with previously reported initial growth rate of ∼600 μm min^−1^ through gas heating modulation on a nonporous substrate, our estimated initial growth rate was estimated to exceed 1 mm min^−1^ (∼1110 μm min^−1^). This data is summarized in Table 2.

**Table 2:** SWCNT growth at the optimized growth conditions and open porosity. The total yield including both sides (one is included in parentheses)

Substrate	Forest height (mm)	Total yield (mg cm^−2^)	G/D ratio (front)	Average Growth rate (μm min^−1^)
Mesh	3.47	25.8 (12.7)	7.0	355
Si	0.88	3.2	6.5	70.8

Importantly, by transmission electron microscopy (TEM), the grown CNTs were confirmed to be composed of 95.1% SWCNTs (Fig. 3b and c). These results represent both the highest average and initial growth rates observed for SWCNT forests. In addition, purity analysis of the CNTs by thermogravimetric analysis (TGA) and specific surface analysis by nitrogen adsorption–desorption isotherms showed that the SWCNTs grown on the porous mesh substrates were on par with those grown on nonporous substrates (ESI†). Specifically, the weight decreases at 450 °C in TGA for the nonporous (Si) and porous substrate were 1.5% and 0.7%, respectively. This indicates that both methods yielded highly pure SWCNTs. In addition, the residual impurities, which are composed of catalyst material or catalyst support was estimated to be 0.22% and 0.15%, respectively. Furthermore, the specific surface area estimated by the Brunauer–Emmett–Teller (BET) formulation was estimated to be 1012 and 1180 m^2^ g^−1^, respectively. Note, the ideal specific surface area for a single side of graphene is 1315 m^2^ g^−1^. These high values indicate both that the grown CNTs possess exceptionally high purity as well as high single-wall selectivity in agreement with TEM data.

## 3. Discussion

Taken together, the above results demonstrate advantages in the synthesis of single-wall carbon nanotubes from porous metal substrates. Although included in our results for reference is synthesis using a flat silicon substrate, we have surveyed the literature to compare our results in various aspects of yield and quality.

### 3.1 Single wall selectivity

The single-wall selectivity for synthesis on porous substrates was estimated to be 95.1% be TEM (Fig. 4b and c). This value is comparable to the highest values reported for single wall synthesis reported the literature for flat substrates grown by ethylene, acetylene, and alcohol CVD methods.^6,7,11,12,19,28,29^

### 3.2 Growth kinetics

The growth kinetics, specifically the growth rates, compare well with the highest growth rate synthesis processes reported. Growth rates for SWCNT forests from flat substrates have been reported in terms of (1) an initial growth rate, which is often indicative to a peak growth rate, and (2) an average growth rate. The highest initial growth rates for SWCNTs was reported through the preheating of the reactant gases resulting in initial growth rates up to 715 μm min^−1^ using acetylene, and 620 μm min^−1^ using ethylene.^12,19^ Typically quite high initial growth rates fall into the range of 50–250 μm min^−1^. Therefore, the initial growth rate of ∼1120 μm min^−1^ reported using a porous substrate ranks among the highest. Although initial growth rate is important, maintaining a high average growth rate over the process is important to achieving tall SWCNT forests, thus high yields. The highest reported average growth rate spanning a 5–10 minutes growth time and achieving SWCNT forest height of 1 mm or more was ∼200–250 μm min^−1^ by several groups using either acetylene or ethylene.^6,12,14,19,30,31^ The average growth rate reported herein was estimated to be ∼340 μm min^−1^ over a 10 minutes span, which, again, ranks as the highest for SWCNT forests.

### 3.3 High yield SWCNT synthesis

The tall SWCNT forests as described above combined with the ∼3-fold increase in available catalyst surface area resulted in a total yield of 25.8 mg cm^−2^. For flat substrates, the yield can only increase by increasing the overall height of the SWCNT forest as the substrate area is fixed. Although not all published reports show the calculated yield values, we estimated the values based on typical SWCNT forest density values.^14^ The highest yield SWCNT reported thus far is 10 mm SWCNT by Yasuda *et al.*, which is estimated to be ∼40 mg cm^−2^ in a 50 minutes growth time.^11^ The authors reported that the purity and the SW-selectivity of this forest decreased due to the extended growth time, this is the highest achieved thus far. If we consider high purity, SWCNT forests, the highest reported yield is approximately 10 mg cm^−2^ for a 2.5 mm tall forest in 10 minutes.^6^ Other values fall within a range of 3–7 mg cm^−2^.^7,12,31,32^ Therefore, our reported value of 25.8 mg cm^−2^ represents a significant increase of the highest reported values at the laboratory scale with high purity and high selectivity.

### 3.4 Purity

Another important aspect of CNT synthesis is purity, which comes in the form of both carbon and noncarbon impurities. Using both TGA and nitrogen adsorption purity analysis techniques, the highest reported purity for SWCNTs was 99.98% carbon purity and a specific surface area ∼1250 m^2^ g^−1^.^6,32,33^ Therefore, the reported carbon purity of 99.3% and specific surface area 1180 m^2^ g^−1^ stands among the highest reported.

### 3.5 Substrate material and cost

One final aspect important to comparing this work with previous reports of synthesis on flat nonporous substrates is the use of inexpensive wire mesh. From a cost perspective, metal mesh are about 1/500-times as expensive as silicon wafers, which are compared here. Secondly, as presented above the high SW-selectivity, high purity, high growth rate were all demonstrated on the same mesh and compared well against the highest reported values for silicon substrates, which was shown to be difficult to achieve.^34^ The results presented here show that by simply changing the substrate from a flat nonporous substrate to porous substrates, as represented by metal meshes, the growth yield and efficiency can be improved without any sacrifice to the fraction of SWCNTs and the overall purity.

## 4. Conclusions

In this study, we report the examination of metal meshes as representative porous substrates and alternatives to nonporous substrates for high efficiency SWCNT synthesis. Our results demonstrate that the synthesis of SWCNT forests is highly dependent on the initial porosity as well as maintaining the porosity within the growth time. Our results demonstrate that CNTs can be grown on all surfaces in high efficiency with an optimum porosity of ∼30%. While our results do show dependence on the wire diameter, aperture size, and relative surface areas compared to flat substrates, porosity and sufficiently large aperture size were required for sustained high speed synthesis. Based on these results, we demonstrated the high efficiency synthesis of SWCNT forests (height: 3.47 mm, average growth rate: 301 μm min^−1^, and yield: 12.7 mg cm^−2^ in a 10 min growth time). In addition, the initial growth rate was found exceed 1 mm per minute. We believe this represents an interesting alternative approach for the efficient synthesis of tall and high yield SWCNT synthesis for mass production through simply a change to metal meshes.

## Conflicts of interest

There are no conflicts to declare.

## Supplementary Material

RA-008-C7RA13093G-s001
