# Supplementary material for: One millimeter per minute growth rates for single wall carbon nanotube forests enabled by porous metal substrates
Source: RSC Adv. 2018 Feb 19;8(14):7810–7. doi: 10.1039/c7ra13093g (PMC9078519; doi:10.1039/c7ra13093g)
Supplement: RA-008-C7RA13093G-s001 [file RA-008-C7RA13093G-s001.pdf]

Electronic Supplementary Material (ESI) for RSC Advances.
This journal is © The Royal Society of Chemistry 2018

Supplemental data

The mesh aperture and opening porosity in Table 1 were calculated in the below equations;

$$Aperature(mm) = \left(\frac{25.4}{M}\right) - d \quad \text{.........(1)}$$

$$Porosity(\%) = \frac{A}{(A+d)} \times 100 \quad \text{.......(2)}$$

, where A is the mesh size (mm), M is the mesh number (wire number / inch), and d is the wire diameter. The specifications of the mesh substrate in this work are summarized in Table 1.

Supplemental Figure

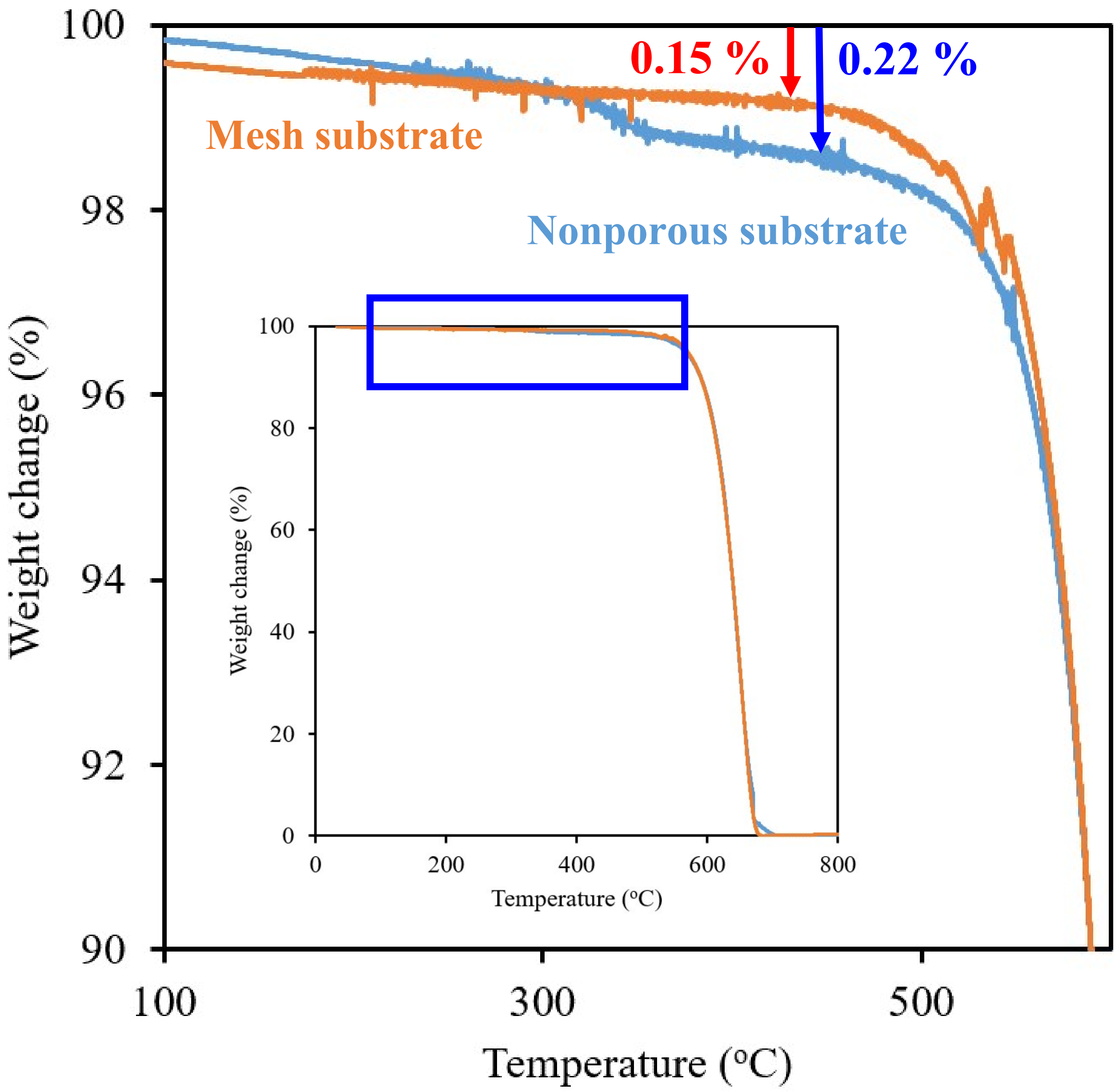

Figure S1 TGA curves of CNTs on mesh and nonporous flat substrate.
